# Supplementary material for: Eukaryotic Genomes Show Strong Evolutionary Conservation of k-mer Composition and Correlation Contributions between Introns and Intergenic Regions
Source: Genes (Basel). 2021 Oct 1;12(10):1571. doi: 10.3390/genes12101571 (PMC8536142; doi:10.3390/genes12101571)
Supplement: Supplementary file 1 [file genes-12-01571-s001.zip › genes-1318773-supplementary.pdf]

Table S1. Organisms and Chromosomes (order of *Embryophyta, Protista, Fungi and Animalia*) used in our analysis (accession numbers of sequences are found within Table S1B), blue are the new genomes in *Animalia* in comparison to previous research

Species	Further Classification	Chromosomes
Physcomitrella patens	Bryophyta	1-27
Brachypodium distachyon	Tracheophyta—Liliopsida	1-5
Oryza sativa	Tracheophyta—Liliopsida	1-12
Zea mays	Tracheophyta—Liliopsida	1-10
Sorghum bicolor	Tracheophyta—Liliopsida	1-10
Setaria italic	Tracheophyta—Liliopsida	I-IX
Ananas comosus	Tracheophyta—Liliopsida	1-25
Elaeis guineensis	Tracheophyta—Liliopsida	1-16
Musa acuminate	Tracheophyta—Liliopsida	1-11
Asparagus officinalis	Tracheophyta—Liliopsida	1-10
Papaver somniferum	Tracheophyta—Ranunculales	1-11
Vitis vinifera	Tracheophyta—Eudicotyledons	1-19
Arabidopsis thaliana	Tracheophyta—Eudicotyledons	1-5
Brassica napus	Tracheophyta—Eudicotyledons	A1-A10, C1-C9
Camelina sativa	Tracheophyta—Eudicotyledons	1-20
Gossypium raimondii	Tracheophyta—Eudicotyledons	1-13
Theobroma cacao	Tracheophyta—Eudicotyledons	1-10
Citrus sinensis	Tracheophyta—Eudicotyledons	1-9
Populus trichocarpa	Tracheophyta—Eudicotyledons	1-19
Manihot esculenta	Tracheophyta—Eudicotyledons	lg1-lg18
Arachis duranensis	Tracheophyta—Eudicotyledons	A1-A10
Phaseolus vulgaris	Tracheophyta—Eudicotyledons	1-11
Cajanus cajan	Tracheophyta—Eudicotyledons	1-11
Vigna angularis	Tracheophyta—Eudicotyledons	1-11
Medicago truncatula	Tracheophyta—Eudicotyledons	1-8
Lupinus angustifolius	Tracheophyta—Eudicotyledons	lg1-lg20
Fragaria vesca	Tracheophyta—Eudicotyledons	lg1-lg7
Malus domestica	Tracheophyta—Eudicotyledons	1-17
Prunus mume	Tracheophyta—Eudicotyledons	lg1-lg8
Cucumis sativus	Tracheophyta—Eudicotyledons	1-7
Beta vulgaris	Tracheophyta—Eudicotyledons	1-9
Nicotiana attenuata	Tracheophyta—Eudicotyledons	1-12
Sesamum indicum	Tracheophyta—Eudicotyledons	lg1-lg16
Helianthus annuus	Tracheophyta—Eudicotyledons	1-17
Dictyostelium discoideum	Amoebozoa	1-6
Leishmania donovani	Euglenozoa	1-36
Leishmania infantum	Euglenozoa	1-36
Leishmania major	Euglenozoa	1-36
Trypanosoma brucei	Euglenozoa	1-11
Neospora caninum	Alveolata	Ia, Ib, II-VI, VIIa, VIIb, VIII-XII
Toxoplasma gondii	Alveolata	Ia, Ib, II-VI, VIIa, VIIb, VIII-XII
Cryptosporidium parvum	Alveolata	1-8
Plasmodium vivax	Alveolata	1-14
Plasmodium falciparum	Alveolata	1-14
Plasmodium berghei	Alveolata	1-14
Plasmodium cynomolgi	Alveolata	1-14
Plasmodium coatneyi	Alveolata	1-14
Plasmodium gaboni	Alveolata	1-14
Theileria annulata	Alveolata	1-4
Theileria parva	Alveolata	1-2
Theileria orientalis	Alveolata	1-4
Babesia microti	Alveolata	I-IV
Babesia bigemina	Alveolata	I-V
Paramecium tetraurelia	Alveolata	MN
Phaeodactylum tricornutum	Stramenopiles	1-33
Ectocarpus siliculosus	Stramenopiles	lg1-lg34
Thalassiosira pseudonana	Stramenopiles	1-10, 12-15, 17, 18, 20, 22-24
Nannochloropsis gaditana	Stramenopiles	1-21
Cyanidioschyzon merolae	Rhodophyta	1-20

Chlamydomonas reinhardtii	Chlorophyta	1-17
Micromonas commoda	Chlorophyta	1-17
Bathycoccus prasinos	Chlorophyta	1-19
Ostreococcus lucimarinus	Chlorophyta	1-21
Ostreococcus tauri	Chlorophyta	1-20
Encephalitozoon cuniculi	Microsporidia	I-XI
Encephalitozoon intestinalis	Microsporidia	I-XI
Ustilago maydis	Basidiomycota—Ustilaginomycetes	1-23
Sporisorium reilianum	Basidiomycota—Ustilaginomycetes	1-23
Cryptococcus gattii	Basidiomycota—Tremellomycetes	A-N
Pyrrhoderma noxium	Basidiomycota—Agaricomycetes	1-12
Schizosaccharomyces pombe	Ascomycota—Schizosaccharomycetes	I-III
Saccharomyces cerevisiae	Ascomycota—Saccharomycetes	I-XVI
Tetrapisispora phaffii	Ascomycota—Saccharomycetes	1-16
Tetrapisispora blattae	Ascomycota—Saccharomycetes	1-10
Zygosaccharomyces rouxii	Ascomycota—Saccharomycetes	A-G
Naumovozyma castellii	Ascomycota—Saccharomycetes	1-10
Lachancea thermotolerans	Ascomycota—Saccharomycetes	A-H
Eremothecium gossypii	Ascomycota—Saccharomycetes	I-VII
Candida glabrata	Ascomycota—Saccharomycetes	A-M
Candida albicans	Ascomycota—Saccharomycetes	1-7, R
Debaryomyces hansenii	Ascomycota—Saccharomycetes	A-G
Komagataella phaffii	Ascomycota—Saccharomycetes	1-4
Yarrowia lipolytica	Ascomycota—Saccharomycetes	A-F
Pichia kudriavzevii	Ascomycota—Saccharomycetes	1-5
Ogataea parapolymorpha	Ascomycota—Saccharomycetes	I-VII
Zymoseptoria tritici	Ascomycota—Dothideomycetes	1-21
Neurospora crassa	Ascomycota—Sordariomycetes	I-VII
Thermothelomyces thermophila	Ascomycota—Sordariomycetes	1-7
Thielavia terrestris	Ascomycota—Sordariomycetes	1-6
Fusarium oxysporum	Ascomycota—Sordariomycetes	1-15
Aspergillus nidulans	Ascomycota—Eurotiomycetes	I-VIII
Aspergillus fumigatus	Ascomycota—Eurotiomycetes	1-8
Penicillium chrysogenum	Ascomycota—Eurotiomycetes	I-IV
Homo sapiens	Mammalia	1–22, X, Y
Pan troglodytes	Mammalia	1, 2A, 2B, 3-22, X, Y
Mus musculus	Mammalia	1–19, X, Y
Rattus norvegicus	Mammalia	1–20, X, Y
Oryctolagus cuniculus	Mammalia	1–21, X
Canis lupus	Mammalia	1–38, X
Felis catus	Mammalia	A1–A3, B1–B4, C1, C2, D1–D4, E1–E3, F1, F2, X
Equus caballus	Mammalia	1–31, X
Capra hircus	Mammalia	1–29
Bos taurus	Mammalia	1–29, X
Sus scrofa	Mammalia	1–18, X, Y
Monodelphis domestica	Mammalia	1–8, X
Ornithorhynchus anaticus	Mammalia	1–7, 10–12, 14, 15, 17, 18, 20, X1, X2, X3, X5
Gallus gallus	Aves	1–28, 30, 33,W, Z
Taeniopygia guttata	Aves	1, 1A, 1B, 2-4, 4A, 5–15, 17–28, Z
Ficedula albicollis	Aves	1–15, 17–28, 1A, 4A, Z
Strigops habroptila	Aves	1-23, W, Z
Falco rusticolus	Aves	1-22. W, Z
Aquila chrysaetos chrysaetos	Aves	1-26, W, Z
Calypte anna	Aves	1-4, 4A, 4B, 5, 5B, 6-15, 17-28, 33, W, Z
Chrysemys picta	Archelosauria	1–11, 13, 15, 17, 19, 21, 22, 24, 25
Anolis carolinensis	Lepidosauria	1–6, a, b, c, d, f, g, h
Lacerta agilis	Lepidosauria	1-18, W, Z
Xenopus tropicalis	Amphibia	1–10
Rana temporaria	Amphibia	1-13
Geotrypetes seraphini	Amphibia	1-19
Takifugu rubripes	Actinopterygii	1–22
Cynoglossus semilaevis	Actinopterygii	1–20,W, Z
Oreochromis niloticus	Actinopterygii	1, 2, 3a, 3b, 4–22
Poecilia reticulata	Actinopterygii	1–23
Danio rerio	Actinopterygii	1–25
Salmo salar	Actinopterygii	1–29

Lepisosteus oculatus	Actinopterygii	1–29
Asterias rubens	Echinodermata	1-22
Ciona intestinalis	Tunicata	1–14
Schistosoma mansoni	Spiralia	1–7,W
Strongyloides ratti	Ecdysozoa	1, 2, X
Caenorhabditis brigsae	Ecdysozoa	1–5, X
Caenorhabditis elegans	Ecdysozoa	1–5, X
Gigantopelta aegis	Mollusca	1-15
Pomacea canaliculata	Mollusca	LG1-LG14
Pecten maximus	Mollusca	1-19
Crassostrea virginica	Mollusca	1-10
Octopus sinensis	Mollusca	LG1-LG30
Lepeophtheirus salmonis	Crustacea	1-15
Penaeus monodon	Crustacea	1-44
Rhipicephalus sanguineus	Chelicerata	1-10
Rhopalosiphum maidis	Insecta	1-4
Apis mellifera	Insecta	1–16
Bombus terrestris	Insecta	1–18
Nasonia vitripennis	Insecta	1–5
Anopheles gambiae	Insecta	2L, 3L, 2R, 3R, X
Drosphila pseudoobscura	Insecta	2, 3
Drosophila melanogaster	Insecta	2L, 2R, 3L, 3R, 4, X, Y
Drosohpila simulans	Insecta	2L, 2R, 3L, 3R, 4, X
Aricia agestis	Insecta	1-22,Z

Table S2. Accession Numbers of Sequences.

Species	Accession Numbers
Physcomitrella patens	NC 037253.1 - NC 037279.1
Brachypodium distachyon	NC 016131.3 - NC 016135.3
Oryza sativa	NC 029256.1 - NC 029267.1
Zea mays	NC 024459.2 - NC 024468.2
Sorghum bicolor	NC 012870.2 - NC 012879.2
Setaria italica	NC 028450.1 - NC 028458.1
Ananas comosus	NC 033621.1 - NC 033645.1
Elaeis guineensis	NC 025993.1 - NC 026008.1
Musa acuminate	NC 025202.1 - NC 025212.1
Asparagus officinalis	NC 033794.1 - NC 033803.1
Papaver somniferum	NC 039358.1 - NC 039368.1
Vitis vinifera	NC 012007.3 - NC 012025.3
Arabidopsis thaliana	NC 003070.9, NC 003071.7, NC 003074.8, NC 003075.7, NC 003076.8
Brassica napus	NC 027757.2 - NC 027775.2
Camelina sativa	NC 025685.1 - NC 025704.1
Gossypium raimondii	NC 026929.1 - NC 026941.1
Theobroma cacao	NC 030850.1 - NC 030859.1
Citrus sinensis	NC 023046.1 - NC 023054.1
Populus trichocarpa	NC 037285.1 - NC 037303.1
Manihot esculenta	NC 035161.1 - NC 035178.1
Arachis duranensis	NC 029772.2 - NC 029781.2
Phaseolus vulgaris	NC 023759.1 - NC 023749.1
Cajanus cajan	NC 033804.1 - NC 033814.1
Vigna angularis	NC 030637.1 - NC 030647.1
Medicago truncatula	NC 016407.2 - NC 016414.2
Lupinus angustifolius	NC 032009.1 - NC 032028.1
Fragaria vesca	NC 020491.1 - NC 020497.1
Malus domestica	NC 024239.1 - NC 024255.1
Prunus mume	NC 024126.1 - NC 024133.1
Cucumis sativus	NC 026655.1 - NC 026661.1
Beta vulgaris	NC 025812.2 - NC 025820.2

Nicotiana attenuate	NC 031989.1 - NC 032000.1
Sesamum indicum	NC 026145.1 - NC 026160.1
Helianthus annuus	NC 035433.1 - NC 035449.1
Dictyostelium discoideum	NC 007087.3, NC 007088.5, NC 007089.4, NC 007090.3, NC 007091.3, NC 007092.3
Leishmania donovani	NC 018228.1 - NC 018263.1
Leishmania infantum	NC 009386.2 - NC 009390.2, NC 009277.2, NC 009391.2 - NC 009420.2
Leishmania major	NC 001905.3, NC 007244.2, NC 004916.2, NC 007245.2 - NC 007273.2, NC 007285.2, NC 007286.2, NC 007284.2, NC 007287.2
Trypanosoma brucei	NC 026734.1 - NC 026744.1
Neospora caninum	NC 018385.1 - NC 018389.1, NC 018391.1 - NC 018395.1, NC 018390.1, NC 018396.1 - NC 018398.1
Toxoplasma gondii	NC 031467.1 - NC 031480.1
Cryptosporidium parvum	NC 006980.1 - NC 006987.1
Plasmodium vivax	NC 009906.1, NC 009907.1, NC 009908.2, NC 009909.1 - NC 009919.1
Plasmodium falciparum	NC 004325.2, NC 037280.1, NC 000521.4, NC 004318.2, NC 004326.2, NC 004327.3, NC 004328.3, NC 004329.3, NC 004330.2, NC 037281.1, NC 037282.1, NC 037284.1, NC _004331.3, NC 037283.1
Plasmodium berghei	NC 036159.1 - NC 036172.1
Plasmodium cynomolgi	NC 020396.1, NC 020395.1, NC 020397.1, NC 020408.1, NC 020398.1 - NC 020407.1
Plasmodium coatneyi	NC 033556.1 - NC 033569.1
Plasmodium gaboni	NC 031481.1 - NC 031494.1
Theileria annulata	NW 001091929.1, NC 011099.1, NC 011100.1, NC 011098.1
Theileria parva	NC 007344.1, NC 007345.1
Theileria orientalis	NC 025260.1 - NC 025263.1
Babesia microti	NC 027205.1, NC 027206.1, NC 027207.2, NC 034969.1
Babesia bigemina	NC 027216.1 - NC 027220.1
Paramecium tetraurelia	NC 006058.1
Phaeodactylum tricornutum	NC 011669.1 - NC 011701.1
Ectocarpus siliculosus	FN649726.1 - FN649759.1
Thalassiosira pseudonana	NC 012064.1 - NC 012073.1, NC 012075.1 - NC 012078.1, NC 012080.1, NC 012081.1, NC 012083.1, NC 012085.1 - NC 012087.1
Nannochloropsis gaditana	CM002455.1 - CM002475.1
Cyanidioschyzon merolae	NC 010127.1 - NC 010146.1
Chlamydomonas reinhardtii	CM008962.1 - CM008978.1
Micromonas commoda	NC 013038.1 - NC 013054.1
Bathycoccus prasinos	NC 024008.1 - NC 023990.1
Ostreococcus lucimarinus	NC 009355.1 - NC 009375.1
Ostreococcus tauri	NC 014426.2 - NC 014445.2
Encephalitozoon cuniculi	NC 003242.2, NC 003229.1 - NC 003235.1, NC 003238.2, NC 003236.1, NC 003237.1
Encephalitozoon intestinalis	NC 014415.1 - NC 014425.1
Ustilago maydis	NC 026478.1 - NC 026500.1
Sporisorium reilianum	FQ311430.1, FQ311441.1, FQ311452.1, FQ311463.1, FQ311470.1 - FQ311474.1, FQ311431.1 - FQ311440.1, FQ311442.1 - FQ311445.1
Cryptococcus gattii	NC 014938.1 - NC 014951.1
Pyrrhoderma noxium	CM008251.1 - CM008262.1
Schizosaccharomyces pombe	NC 003424.3, NC 003423.3, NC 003421.2
Saccharomyces cerevisiae	NC 001133.9, NC 001134.8, NC 001135.5, NC 001136.10, NC 001137.3, NC 001138.5, NC 001139.9, NC 001140.6, NC 001141.2, NC 001142.9, NC 001143.9, NC 001144.5, NC 001145.3, NC _001146.8, NC 001147.6, NC _001148.4
Tetrapisispora phaffii	NC 016520.1 - NC 016535.1
Tetrapisispora blattae	NC 020185.1 - NC 020194.1
Zygosaccharomyces rouxii	NC 012990.1 - NC 012996.1
Naumovozyma castellii	NC 016491.1 - NC 016500.1

Lachancea thermotolerans	NC 013077.1 - NC 013084.1
Eremothecium gossypii	NC 005782.2, NC 005783.5, NC 005784.3, NC 005785.6, NC 005786.2, NC 005787.5, NC 005788.4
Candida glabrata	NC 005967.2, NC 005968.1, NC 006026.1, NC 006027.1, NC 006028.2, NC 006029.1, NC 006030.1, NC 006031.1, NC 006032.2, NC 006033.2, NC 006034.2, NC 006035.2, NC 006036.2
Candida albicans	NC 032089.1 - NC 032096.1
Debaryomyces hansenii	NC 006043.2 - NC 006049.2
Komagataella phaffii	NC 012963.1 - NC 012966.1
Yarrowia lipolytica	NC 006067.1 - NC 006072.1
Pichia kudriavzevii	CP028773.1 - CP028777.1
Ogataea parapolymorpha	NC 027866.1 - NC 027860.1
Zymoseptoria tritici	NC 018218.1 - NC 018198.1
Neurospora crassa	NC 026501.1, NC 026502.1, NC 026503.1, NC 026504.1, NC 026505.1, NC 026506.1, NC 026507.1
Thermothelomyces thermophila	NC 016472.1, NC 016473.1, NC 016474.1, NC 016475.1, NC 016476.1, NC 016477.1, NC 016478.1
Thielavia terrestris	NC 016457.1, NC 016458.1, NC 016459.1, NC 016460.1, NC _016461.1, NC 016462.1
Fusarium oxysporum	NC 030986.1 - NC 031000.1
Aspergillus nidulans	BN001301.1 - BN001308.1
Aspergillus fumigatus	NC 007194.1 - NC 007201.1
Penicillium chrysogenum	CM002798.1 - CM002801.1
Homo sapiens	NC 000001.11, NC 000002.12, NC 000003.12, NC 000004.12, NC 000005.10, NC 000006.12, NC 000007.14, NC 000008.11, NC 000009.12, NC 000010.11, NC 000011.10, NC 000012.12, NC 000013.11, NC 000014.9, NC 000015.10, NC 000016.10, NC 000017.11, NC 000018.10, NC 000019.10, NC 000020.11, NC 000021.9, NC 000022.11, NC 000023.11, NC 000024.10
Pan troglodytes	NC 006468.4 - NC 006492.4
Mus musculus	NC 000067.6 - NC 000087.7
Rattus norvegicus	NC 005100.4 - NC 005120.4, NC 024475.1
Oryctolagus cuniculus	NC 013669.1 - NC 013690.1
Canis lupus	NC 006583.3, NC 006584.3, NC 006585.3, NC 006586.3, NC 006587.3, NC 006588.3, NC 006589.3, NC 006590.3, NC 006591.3, NC 006592.3, NC 006593.3, NC 006594.3, NC 006595.3, NC 006596.3, NC 006597.3, NC 006598.3, NC 006599.3, NC 006600.3, NC 006601.3, NC 006602.3, NC 006603.3, NC 006604.3, NC 006605.3, NC 006606.3, NC 006607.3, NC 006608.3, NC 006609.3, NC 006610.3, NC 006611.3, NC 006612.3, NC 006613.3, NC 006614.3, NC 006615.3, NC 006616.3, NC 006617.3, NC 006618.3, NC 006619.3, NC 006620.3, NC 006621.3
Felis catus	NC 018723.2, NC 018724.2, NC 018725.2, NC 018726.2, NC 018727.2, NC 018728.2, NC 018729.2, NC 018730.2, NC 018731.2, NC 018732.2, NC 018733.2, NC 018734.2, NC 018735.2, NC 018736.2, NC 018737.2, NC 018738.2, NC 018739.2, NC 018740.2, NC 018741.2
Equus caballus	NC 009144.2, NC 009145.2, NC 009146.2, NC 009147.2, NC 009148.2, NC 009149.2, NC 009150.2, NC 009151.2, NC 009152.2, NC 009153.2, NC 009154.2, NC 009155.2, NC 009156.2, NC 009157.2, NC 009158.2, NC 009159.2, NC 009160.2, NC 009161.2, NC 009162.2, NC 009163.2, NC 009164.2, NC 009165.2, NC 009166.2, NC 009167.2, NC 009168.2, NC 009169.2, NC 009170.2, NC 009171.2, NC 009172.2, NC 009173.2, NC 009174.2, NC 009175.2
Capra hircus	NC 030808.1, NC 030809.1, NC 030810.1, NC 030811.1, NC 030812.1, NC 030813.1, NC 030814.1, NC 030815.1, NC 030816.1, NC 030817.1, NC 030818.1, NC 030819.1, NC 030820.1, NC 030821.1, NC 030822.1, NC 030823.1, NC 030824.1, NC 030825.1, NC 030826.1, NC 030827.1, NC 030828.1, NC 030829.1, NC 030830.1, NC 030831.1, NC 030832.1, NC 030833.1, NC 030834.1, NC 030835.1, NC 030836.1

Bos taurus	AC 000158.1, AC 000159.1, AC 000160.1, AC 000161.1, AC 000162.1, AC 000163.1, AC 000164.1, AC 000165.1, AC 000166.1, AC 000167.1, AC 000168.1, AC 000169.1, AC 000170.1, AC 000171.1, AC 000172.1, AC 000173.1, AC 000174.1, AC 000175.1, AC 000176.1, AC 000177.1, AC 000178.1, AC 000179.1, AC 000180.1, AC 000181.1, AC 000182.1, AC 000183.1, AC 000184.1, AC 000185.1, AC 000186.1, AC 000187.1
Sus scrofa	NC 010443.5, NC 010444.4, NC 010445.4, NC 010446.5, NC 010447.5, NC 010448.4, NC 010449.5, NC 010450.4, NC 010451.4, NC 010452.4, NC 010453.5, NC 010454.4, NC 010455.5, NC 010456.5, NC 010457.5, NC 010458.4, NC 010459.5, NC 010460.4, NC 010461.5, NC 010462.3
Monodelphis domestica	NC 008801.1, NC 008802.1, NC 008803.1, NC 008804.1, NC 008805.1, NC 008806.1, NC 008807.1, NC 008808.1, NC 008809.1
Ornithorhynchus anaticus	NC 009094.1, NC 009095.1, NC 009096.1, NC 009097.1, NC 009098.1, NC 009099.1, NC 009100.1, NC 009103.1, NC 009104.1, NC 009105.1, NC 009107.1, NC 009108.1, NC 009110.1, NC 009111.1, NC 009112.1, NC 009114.1, NC 009115.1, NC 009116.1, NC 009118.1
Gallus gallus	NC 006088.4, NC 006089.4, NC 006090.4, NC 006091.4, NC 006092.4, NC 006093.4, NC 006094.4, NC 006095.4, NC 006096.4, NC 006097.4, NC 006098.4, NC 006099.4, NC 006100.4, NC 006101.4, NC 006102.4, NC 006103.4, NC 006104.4, NC 006105.4, NC 006106.4, NC 006107.4, NC 006108.4, NC 006109.4, NC 006110.4, NC 006111.4, NC 006112.3, NC 006113.4, NC 006114.4, NC 006115.4, NC 028739.1, NC 028740.1, NC 006119.3, NC 008465.3, NC 006126.4, NC 006127.4
Taeniopygia guttata	NC 011462.1, NC 011463.1, NC 011464.1, NC 011465.1, NC 011466.1, NC 011467.1, NC 011468.1, NC 011469.1, NC 011470.1, NC 011471.1, NC 011472.1, NC 011473.1, NC 011474.1, NC 011475.1, NC 011476.1, NC 011477.1, NC 011478.1, NC 011479.1, NC 011480.1, NC 011481.1, NC 011482.1, NC 011483.1, NC 011484.1, NC 011485.1, NC 011486.1, NC 011487.1, NC 011488.1, NC 011489.1, NC 011490.1, NC 011491.1, NC 011492.1, NC 011496.1, NC 011494.1, NC 011495.1, NC 011493.1
Ficedula albicollis	NC 021671.1, NC 021672.1, NC 021673.1, NC 021674.1, NC 021675.1, NC 021676.1, NC 021677.1, NC 021678.1, NC 021679.1, NC 021680.1, NC 021681.1, NC 021682.1, NC 021683.1, NC 021684.1, NC 021685.1, NC 021686.1, NC 021687.1, NC 021688.1, NC 021689.1, NC 021690.1, NC 021691.1, NC 021692.1, NC 021693.1, NC 021694.1, NC 021695.1, NC 021696.1, NC 021697.1, NC 021698.1, NC 021699.1, NC 021700.1
Strigops habroptila	NC 044277.2 - NC 044279.2, NC 044281.2 - NC 044285.2, NC 044288.2 - NC 044299.2, NC 044301.2 - NC 044302.2, NC 046358.1 - NC 046360.1
Falco rusticolus	NC 051187.1 - NC 051210.1
Aquila chrysaetos chrysaetos	NC 044004.1 - NC 044030.1, NC 054457.1
Calypte anna	NC 044244.1 - NC 044276.1
Chrysemys picta	NC 024218.1, NC 024219.1, NC 024220.1, NC 024221.1, NC 024222.1, NC 024223.1, NC 024224.1, NC 024225.1, NC 024226.1, NC 024227.1, NC 024228.1, NC 024229.1, NC 024230.1, NC 024231.1, NC 024232.1, NC 024233.1, NC 024234.1, NC 024235.1
Anolis carolinensis	NC 014776.1, NC 014777.1, NC 014778.1, NC 014779.1, NC 014780.1, NC 014781.1, NC 014782.1, NC 014783.1, NC 014784.1, NC 014785.1, NC 014786.1, NC 014787.1, NC 014788.1
Lacerta agilis	NC 046312.1 - NC 046331.1
Xenopus tropicalis	NC 030677.1, NC 030678.1, NC 030679.1, NC 030680.1, NC 030681.1, NC 030682.1, NC 030683.1, NC 030684.1, NC 030685.1, NC 030686.1
Rana temporaria	NC 053489.1 - NC 053501.1
Geotrypetes seraphini	NC 047084.1 - NC 047102.1
Takifugu rubripes	NC 018890.1, NC 018891.1, NC 018892.1, NC 018893.1, NC 018894.1, NC 018895.1, NC 018896.1, NC 018897.1, NC 018898.1, NC 018899.1, NC 018900.1, NC 018901.1, NC 018902.1, NC 018903.1, NC 018904.1, NC 018905.1, NC 018906.1, NC 018907.1, NC 018908.1, NC 018909.1, NC 018910.1, NC 018911.1

Cynoglossus semilaevis	NC 024307.1, NC 024308.1, NC 024309.1, NC 024310.1, NC 024311.1, NC 024312.1, NC 024313.1, NC 024314.1, NC 024315.1, NC 024316.1, NC 024317.1, NC 024318.1, NC 024319.1, NC 024320.1, NC 024321.1, NC 024322.1, NC 024323.1, NC 024324.1, NC 024325.1, NC 024326.1, NC 024327.1, NC 024328.1
Oreochromis niloticus	NC 031965.1, NC 031966.1, NC 031967.1, NC 031968.1, NC 031969.1, NC 031970.1, NC 031971.1, NC 031972.1, NC 031973.1, NC 031974.1, NC 031975.1, NC 031976.1, NC 031977.1, NC 031978.1, NC 031979.1, NC 031980.1, NC 031987.1, NC 031981.1, NC 031982.1, NC 031983.1, NC 031984.1, NC 031985.1, NC 031986.1
Poecilia reticulata	NC 024331.1, NC 024332.1, NC 024333.1, NC 024334.1, NC 024335.1, NC 024336.1, NC 024337.1, NC 024338.1, NC 024339.1, NC 024340.1, NC 024341.1, NC 024342.1, NC 024343.1, NC 024344.1, NC 024345.1, NC 024346.1, NC 024347.1, NC 024348.1, NC 024349.1, NC 024350.1, NC 024351.1, NC 024352.1, NC 024353.1
Danio rerio	NC 007112.6, NC 007113.6, NC 007114.6, NC 007115.6, NC 007116.6, NC 007117.6, NC 007118.6, NC 007119.6, NC 007120.6, NC 007121.6, NC 007122.6, NC 007123.6, NC 007124.6, NC 007125.6, NC 007126.6, NC 007127.6, NC 007128.6, NC 007129.6, NC 007130.6, NC 007131.6, NC 007132.6, NC 007133.6, NC 007134.6, NC 007135.6, NC 007136.6
Salmo salar	NC 027300.1, NC 027301.1, NC 027302.1, NC 027303.1, NC 027304.1, NC 027305.1, NC 027306.1, NC 027307.1, NC 027308.1, NC 027309.1, NC 027310.1, NC 027311.1, NC 027312.1, NC 027313.1, NC 027314.1, NC 027315.1, NC 027316.1, NC 027317.1, NC 027318.1, NC 027319.1, NC 027320.1, NC 027321.1, NC 027322.1, NC 027323.1, NC 027324.1, NC 027325.1, NC 027326.1, NC 027327.1, NC 027328.1
Lepisosteus oculatus	NC 023179.1, NC 023180.1, NC 023181.1, NC 023182.1, NC 023183.1, NC 023184.1, NC 023185.1, NC 023186.1, NC 023187.1, NC 023188.1, NC 023189.1, NC 023190.1, NC 023191.1, NC 023192.1, NC 023193.1, NC 023194.1, NC 023195.1, NC 023196.1, NC 023197.1, NC 023198.1, NC 023199.1, NC 023200.1, NC 023201.1, NC 023202.1, NC 023203.1, NC 023204.1, NC 023205.1, NC 023206.1, NC 023207.1
Asterias rubens	NC 047062.1 - NC 047083.1
Ciona intestinalis	NC 020166.2, NC 020167.2, NC 020168.2, NC 020169.2, NC 020170.2, NC 020171.2, NC 020172.2, NC 020173.2, NC 020174.2, NC 020175.2, NC 020176.2, NC 020177.2, NC 020178.2, NC 020179.2
Schistosoma mansoni	NC 031496.1, NC 031497.1, NC 031498.1, NC 031499.1, NC 031500.1, NC 031501.1
Strongyloides ratti	LN609528.1, LN609529.1, LN609530.1
Caenorhabditis brigsae	FR847112.2, FR847113.2, FR847114.2, FR847118.2, FR847121.2, FR847123.2
Caenorhabditis elegans	NC 003279.8, NC 003280.10, NC 003281.10, NC 003282.8, NC 003283.11, NC 003284.9
Gigantopelta aegis	NC 054699.1 - NC 054713.1
Pomacea canaliculata	NC 037590.1 - NC 037603.1
Pecten maximus	NC 047015.1- NC 047033.1
Crassostrea virginica	NC 035780.1 - NC 035789.1
Octopus sinensis	NC 042997.1 - NC 043026.1
Lepeophtheirus salmonis	NC 052131.1 - NC 052145.1
Penaeus monodon	NC 051386.1 - NC 051429.1
Rhipicephalus sanguineus	NC 051176.1 - NC 051186.1
Rhopalosiphum maidis	NC 040877.1 - NC 040880.1
Apis mellifera	NC 007070.3, NC 007071.3, NC 007072.3, NC 007073.3, NC 007074.3, NC 007075.3, NC 007076.3, NC 007077.3, NC 007078.3, NC 007079.3, NC 007080.3, NC 007081.3, NC 007082.3, NC 007083.3, NC 007084.3, NC 007085.3
Bombus terrestris	NC 015762.1, NC 015763.1, NC 015764.1, NC 015765.1, NC 015766.1, NC 015767.1,

	NC 015768.1, NC 015769.1, NC 015770.1, NC 015771.1, NC 015772.1, NC 015773.1, NC 015774.1, NC 015775.1, NC 015776.1, NC 015777.1, NC 015778.1, NC 015779.1
Nasonia vitripennis	NC 015867.2, NC 015868.2, NC 015869.2, NC 015870.2, NC 015871.2
Anopheles gambiae	NT_078265.2, NT_078267.5, NC 004818.2, NT_078266.2, NT_078268.4
Drosphila pseudoobscura	NC 009005.2, NC 009006.2
Drosophila melanogaster	NC 004354.4, NT_033779.5, NT_033778.4, NT_037436.4, NT_033777.3, NC 004353.4, NC 024512.1
Drosohpila simulans	NT_479533.1, NT_479534.1, NT_479535.1, NT_479536.1, NC 029796.1, NC 029795.1
Aricia agestis	NC 056406.1 - NC 056428.1

Table S3. mean correlation values from spectra (1000 Mbp length, mean over 5 repetitions) simulated by zero order *Markov models*

k	correlation values
1	-0.167 ± 0.167
2	-0.109 ± 0.059
3	-0.070 ± 0.035
4	-0.004 ± 0.017
5	0.044 ± 0.009
6	0.011 ± 0.004
7	0.002 ± 0.004
8	0.0001 ± 0.0015
9	-0.0003 ± 0.0009
10	0.00005 ± 0.00039
11	-0.00003 ± 0.00019

Table S4. reference correlation contribution of tandem repeat words, derived from random spectra generated using first order Markov Models (1000 Mbp length, mean over 5 repetitions)

G+C Content of correlated sequences	missmatches	k	Correlation Contribution b = 1 in %	Correlation Contribution b = 2 in %	Correlation Contribution b = 3 in %	remaining words
50% - 50%	0	7	0.090 ± 0.143	0.139 ± 0.147	0.311 ± 0.280	99.460 ± 0.347
45% - 50%	0	7	0.178 ± 0.095	-0.709 ± 1.899	-0.595 ± 0.454	101.127 ± 4.464
45% - 45%	0	7	0.198 ± 0.001	0.204 ± 0.001	0.845 ± 0.002	98.753 ± 0.002
40% - 50%	0	7	0.716 ± 0.517	-0.697 ± 2.297	-0.495 ± 0.067	100.476 ± 5.305
40% - 45%	0	7	0.235 ± 0.001	0.241 ± 0.001	0.935 ± 0.002	98.589 ± 0.002
40% - 40%	0	7	0.282 ± 0.001	0.289 ± 0.001	1.053 ± 0.001	98.377 ± 0.001
35% - 50%	0	7	0.469 ± 0.228	-0.739 ± 2.940	-0.709 ± 0.044	100.979 ± 6.672
35% - 45%	0	7	0.291 ± 0.001	0.296 ± 0.001	1.078 ± 0.002	98.334 ± 0.003
35% - 40%	0	7	0.348 ± 0.001	0.354 ± 0.001	1.223 ± 0.001	98.075 ± 0.002
35% - 35%	0	7	0.423 ± 0.001	0.429 ± 0.001	1.420 ± 0.001	97.728 ± 0.002
30% - 50%	0	7	0.439 ± 0.175	-0.845 ± 3.798	-0.267 ± 0.391	100.673 ± 8.527
30% - 45%	0	7	0.37 ± 0.002	0.373 ± 0.002	1.282 ± 0.002	97.976 ± 0.003
30% - 40%	0	7	0.434 ± 0.001	0.440 ± 0.001	1.452 ± 0.001	97.673 ± 0.002
30% - 35%	0	7	0.517 ± 0.001	0.522 ± 0.001	1.673 ± 0.001	97.288 ± 0.002
30% - 30%	0	7	0.615 ± 0.001	0.620 ± 0.001	1.943 ± 0.002	96.822 ± 0.002
25% - 50%	0	7	0.647 ± 0.359	-1.029 ± 4.871	-0.192 ± 0.108	100.575 ± 10.872
25% - 45%	0	7	0.473 ± 0.002	0.475 ± 0.002	1.557 ± 0.003	97.494 ± 0.004
25% - 40%	0	7	0.544 ± 0.001	0.550 ± 0.001	1.749 ± 0.002	97.157 ± 0.002

25% - 35%	0	7	0.631 ± 0.001	0.636 ± 0.001	1.985 ± 0.001	96.748 ± 0.002
25% - 30%	0	7	0.730 ± 0.001	0.735 ± 0.001	2.262 ± 0.002	96.273 ± 0.002
25% - 25%	0	7	0.841 ± 0.001	0.846 ± 0.001	2.577 ± 0.002	95.736 ± 0.002
50% - 50%	0	11	0.0001 ± 0.0006	0.0003 ± 0.0005	0.0019 ± 0.0011	99.9977 ± 0.0014
45% - 50%	0	11	-0.0582 ± 0.4376	0.1704 ± 0.3500	0.728 ± 0.5892	99.1598 ± 0.8132
45% - 45%	0	11	0.0017 ± 0.0001	0.0017 ± 0.0001	0.0061 ± 0.0001	99.9905 ± 0.0002
40% - 50%	0	11	0.0170 ± 0.5067	0.2838 ± 0.4537	0.6009 ± 0.6598	99.0982 ± 0.9476
40% - 45%	0	11	0.0025 ± 0.0001	0.0025 ± 0.0001	0.0085 ± 0.0002	99.9865 ± 0.0002
40% - 40%	0	11	0.0036 ± 0.0001	0.0037 ± 0.0001	0.0122 ± 0.0002	99.9806 ± 0.0003
35% - 50%	0	11	0.0566 ± 0.6137	0.3729 ± 0.5592	0.4896 ± 0.7605	99.0809 ± 1.126
35% - 45%	0	11	0.0041 ± 0.0001	0.0040 ± 0.0002	0.0129 ± 0.0002	99.9789 ± 0.0003
35% - 40%	0	11	0.0057 ± 0.0002	0.0058 ± 0.0001	0.0183 ± 0.0003	99.9702 ± 0.0003
35% - 35%	0	11	0.0086 ± 0.0002	0.0086 ± 0.0002	0.0268 ± 0.0003	99.9559 ± 0.0004
30% - 50%	0	11	0.0873 ± 0.7269	0.4638 ± 0.6656	0.4278 ± 0.8886	99.021 ± 1.3271
30% - 45%	0	11	0.0067 ± 0.0002	0.0066 ± 0.0003	0.0202 ± 0.0004	99.9664 ± 0.0005
30% - 40%	0	11	0.0089 ± 0.0003	0.009 ± 0.0002	0.0279 ± 0.0004	99.9542 ± 0.0005
30% - 35%	0	11	0.0128 ± 0.0003	0.0128 ± 0.0002	0.0392 ± 0.0003	99.9353 ± 0.0004
30% - 30%	0	11	0.0178 ± 0.0003	0.018 ± 0.0001	0.0544 ± 0.0003	99.9098 ± 0.0004
25% - 50%	0	11	0.1167 ± 0.8854	0.5827 ± 0.8115	0.4254 ± 1.0778	98.8753 ± 1.6137
25% - 45%	0	11	0.011 ± 0.0003	0.0109 ± 0.0005	0.0325 ± 0.0006	99.9456 ± 0.0009
25% - 40%	0	11	0.0139 ± 0.0004	0.0141 ± 0.0003	0.0429 ± 0.0006	99.9291 ± 0.0008
25% - 35%	0	11	0.0188 ± 0.0003	0.0189 ± 0.0003	0.0574 ± 0.0004	99.9049 ± 0.0006
25% - 30%	0	11	0.0248 ± 0.0003	0.0251 ± 0.0002	0.0754 ± 0.0003	99.8747 ± 0.0005
25% - 25%	0	11	0.0325 ± 0.0003	0.0332 ± 0.0003	0.0989 ± 0.0004	99.8354 ± 0.0006
50% - 50%	1	7	0.639 ± 0.354	1.671 ± 0.565	8.322 ± 1.278	89.368 ± 1.442
45% - 50%	1	7	5.881 ± 5.919	-0.093 ± 6.334	0.773 ± 13.337	93.439 ± 15.907
45% - 45%	1	7	2.830 ± 0.003	3.295 ± 0.003	14.451 ± 0.006	79.425 ± 0.008
40% - 50%	1	7	9.050 ± 6.903	0.156 ± 7.238	1.277 ± 14.612	99.517 ± 17.707
40% - 45%	1	7	3.154 ± 0.003	3.588 ± 0.003	15.268 ± 0.006	77.989 ± 0.007
40% - 40%	1	7	3.551 ± 0.002	3.954 ± 0.002	16.257 ± 0.005	76.238 ± 0.006
35% - 50%	1	7	12.907 ± 8.454	0.316 ± 8.700	1.549 ± 16.764	85.228 ± 20.693
35% - 45%	1	7	3.641 ± 0.004	4.029 ± 0.004	16.501 ± 0.007	75.829 ± 0.009
35% - 40%	1	7	4.100 ± 0.002	4.458 ± 0.002	17.631 ± 0.005	73.81 ± 0.006
35% - 35%	1	7	4.698 ± 0.002	5.014 ± 0.002	19.111 ± 0.004	71.176 ± 0.005
30% - 50%	1	7	7.299 ± 10.501	0.384 ± 10.672	1.486 ± 19.718	90.831 ± 24.758
30% - 45%	1	7	4.294 ± 0.005	4.624 ± 0.005	18.142 ± 0.008	72.94 ± 0.01
30% - 40%	1	7	4.798 ± 0.003	5.103 ± 0.003	19.365 ± 0.005	70.734 ± 0.006
30% - 35%	1	7	5.425 ± 0.002	5.692 ± 0.002	20.898 ± 0.004	67.985 ± 0.005
30% - 30%	1	7	6.158 ± 0.003	6.382 ± 0.002	22.678 ± 0.004	64.782 ± 0.005
25% - 50%	1	7	2.146 ± 13.055	0.343 ± 13.164	1.025 ± 23.498	96.485 ± 29.931
25% - 45%	1	7	5.126 ± 0.006	5.388 ± 0.006	20.201 ± 0.010	69.285 ± 0.013
25% - 40%	1	7	5.653 ± 0.003	5.895 ± 0.003	21.455 ± 0.006	66.997 ± 0.007
25% - 35%	1	7	6.283 ± 0.003	6.494 ± 0.003	22.974 ± 0.005	64.249 ± 0.006
25% - 30%	1	7	6.993 ± 0.003	7.17 ± 0.002	24.677 ± 0.004	61.160 ± 0.005
25% - 25%	1	7	7.778 ± 0.003	7.915 ± 0.002	26.531 ± 0.004	57.776 ± 0.005
50% - 50%	1	11	0.0032 ± 0.0016	0.0101 ± 0.0027	0.0513 ± 0.006	99.9354 ± 0.0068
45% - 50%	1	11	-1.1581 ± 1.6691	0.1526 ± 1.7082	1.3705 ± 3.3783	99.6351 ± 4.1372
45% - 45%	1	11	0.0394 ± 0.0004	0.0412 ± 0.0004	0.1619 ± 0.0007	99.7575 ± 0.0009
40% - 50%	1	11	-1.2876 ± 1.9159	1.1915 ± 1.9523	1.5788 ± 3.6232	98.5174 ± 4.5398
40% - 45%	1	11	0.0549 ± 0.0004	0.0569 ± 0.0004	0.2075 ± 0.0008	99.6808 ± 0.001
40% - 40%	1	11	0.0759 ± 0.0004	0.0783 ± 0.0004	0.2704 ± 0.0007	99.5754 ± 0.0009

35% - 50%	1	11	-1.3811 ± 2.1689	1.8421 ± 2.1778	1.7968 ± 3.9054	97.7423 ± 4.9698
35% - 45%	1	11	0.0815 ± 0.0006	0.0829 ± 0.0006	0.2832 ± 0.0011	99.5525 ± 0.0015
35% - 40%	1	11	0.1100 ± 0.0005	0.112 ± 0.0004	0.3695 ± 0.0008	99.4085 ± 0.0011
35% - 35%	1	11	0.1533 ± 0.0005	0.1544 ± 0.0005	0.4957 ± 0.0009	99.1965 ± 0.0011
30% - 50%	1	11	-1.4292 ± 2.4261	2.3362 ± 2.4303	1.8672 ± 4.274	97.2258 ± 5.4827
30% - 45%	1	11	0.1231 ± 0.001	0.1243 ± 0.001	0.4019 ± 0.0017	99.3506 ± 0.0022
30% - 40%	1	11	0.1603 ± 0.0007	0.1624 ± 0.0006	0.515 ± 0.0012	99.1623 ± 0.0015
30% - 35%	1	11	0.214 ± 0.0006	0.2149 ± 0.0006	0.6718 ± 0.001	98.8994 ± 0.0013
30% - 30%	1	11	0.2848 ± 0.0006	0.2856 ± 0.0006	0.8772 ± 0.001	98.5524 ± 0.0014
25% - 50%	1	11	-1.5242 ± 2.8032	2.8792 ± 2.7997	2.0321 ± 4.8923	96.6129 ± 6.2953
25% - 45%	1	11	0.1859 ± 0.0016	0.1868 ± 0.0016	0.5824 ± 0.0027	99.0449 ± 0.0035
25% - 40%	1	11	0.2320 ± 0.0010	0.2340 ± 0.0009	0.7241 ± 0.0017	98.8098 ± 0.0021
25% - 35%	1	11	0.2954 ± 0.0008	0.2959 ± 0.0007	0.9108 ± 0.0014	98.4978 ± 0.0018
25% - 30%	1	11	0.3749 ± 0.0007	0.3751 ± 0.0007	1.1417 ± 0.0012	98.1083 ± 0.0016
25% - 25%	1	11	0.4700 ± 0.0008	0.4696 ± 0.0007	1.4221 ± 0.0014	97.6384 ± 0.0017

Table S5. Subsets in Protista and Fungi.

Species	Taxonomic Subgroup	Subgroup based on Heatmap
Dictyostelium discoideum	Amoebozoa	#S1 Protista
Leishmania donovani	Euglenozoa	#S2 Protista
Leishmania infantum	Euglenozoa	#S2 Protista
Leishmania major	Euglenozoa	#S2 Protista
Trypanosoma brucei	Euglenozoa	#S1 Protista
Neospora caninum	Alveolata	#S2 Protista
Toxoplasma gondii	Alveolata	#S2 Protista
Cryptosporidium parvum	Alveolata	#S1 Protista
Plasmodium vivax	Alveolata	#S1 Protista
Plasmodium falciparum	Alveolata	#S1 Protista
Plasmodium berghei	Alveolata	#S1 Protista
Plasmodium cynomolgi	Alveolata	#S1 Protista
Plasmodium coatneyi	Alveolata	#S1 Protista
Plasmodium gaboni	Alveolata	#S1 Protista
Theileria annulata	Alveolata	#S1 Protista
Theileria parva	Alveolata	#S1 Protista
Theileria orientalis	Alveolata	#S1 Protista
Babesia microti	Alveolata	#S1 Protista
Babesia bigemina	Alveolata	#S2 Protista
Paramecium tetraurelia	Alveolata	#S1 Protista
Phaeodactylum tricornutum	Stramenopiles	#S2 Protista
Ectocarpus siliculosus	Stramenopiles	#S2 Protista
Thalassiosira pseudonana	Stramenopiles	#S2 Protista
Nannochloropsis gaditana	Stramenopiles	#S2 Protista
Cyanidioschyzon merolae	Rhodopyhta	#S2 Protista
Chlamydomonas reinhardtii	Chlorophyta	#S2 Protista
Micromonas commoda	Chlorophyta	#S2 Protista
Bathycoccus prasinos	Chlorophyta	#S2 Protista
Ostreococcus lucimarinus	Chlorophyta	#S2 Protista
Ostreococcus tauri	Chlorophyta	#S2 Protista
Encephalitozoon cuniculi	Microsporidia	#S2 Fungi
Encephalitozoon intestinalis	Microsporidia	#S2 Fungi
Ustilago maydis	Basidiomycota; Ustilaginomycetes	#S2 Fungi
Sporisorium reilianum	Basidiomycota; Ustilaginomycetes	#S2 Fungi
Cryptococcus gattii	Basidiomycota; Tremellomycetes	#S2 Fungi

Pyrrhoderma noxium	Basidiomycota; Agaricomycetes	#S1 Fungi
Schizosaccharomyces pombe	Ascomycota; Schizosaccharomycetes	#S1 Fungi
Saccharomyces cerevisiae	Ascomycota; Saccharomycetes	#S1 Fungi
Tetrapisispora phaffii	Ascomycota; Saccharomycetes	#S1 Fungi
Tetrapisispora blattae	Ascomycota; Saccharomycetes	#S1 Fungi
Zygosaccharomyces rouxii	Ascomycota; Saccharomycetes	#S1 Fungi
Naumovozyma castellii	Ascomycota; Saccharomycetes	#S1 Fungi
Lachancea thermotolerans	Ascomycota; Saccharomycetes	#S1 Fungi
Eremothecium gossypii	Ascomycota; Saccharomycetes	#S1 Fungi
Candida glabrata	Ascomycota; Saccharomycetes	#S1 Fungi
Candida albicans	Ascomycota; Saccharomycetes	#S1 Fungi
Debaryomyces hansenii	Ascomycota; Saccharomycetes	#S1 Fungi
Komagataella phaffii	Ascomycota; Saccharomycetes	#S1 Fungi
Yarrowia lipolytica	Ascomycota; Saccharomycetes	#S1 Fungi
Pichia kudriavzevii	Ascomycota; Saccharomycetes	#S1 Fungi
Ogataea parapolymorpha	Ascomycota; Saccharomycetes	#S2 Fungi
Zymoseptoria tritici	Ascomycota; Dothideomycetes	#S2 Fungi
Neurospora crassa	Ascomycota; Sordariomycetes	#S2 Fungi
Thermothelomyces thermophila	Ascomycota; Sordariomycetes	#S2 Fungi
Thielavia terrestris	Ascomycota; Sordariomycetes	#S2 Fungi
Fusarium oxysporum	Ascomycota; Sordariomycetes	#S2 Fungi
Aspergillus nidulans	Ascomycota; Eurotiomycetes	#S2 Fungi
Aspergillus fumigatus	Ascomycota; Eurotiomycetes	#S2 Fungi
Penicillium chrysogenum	Ascomycota; Eurotiomycetes	#S2 Fungi

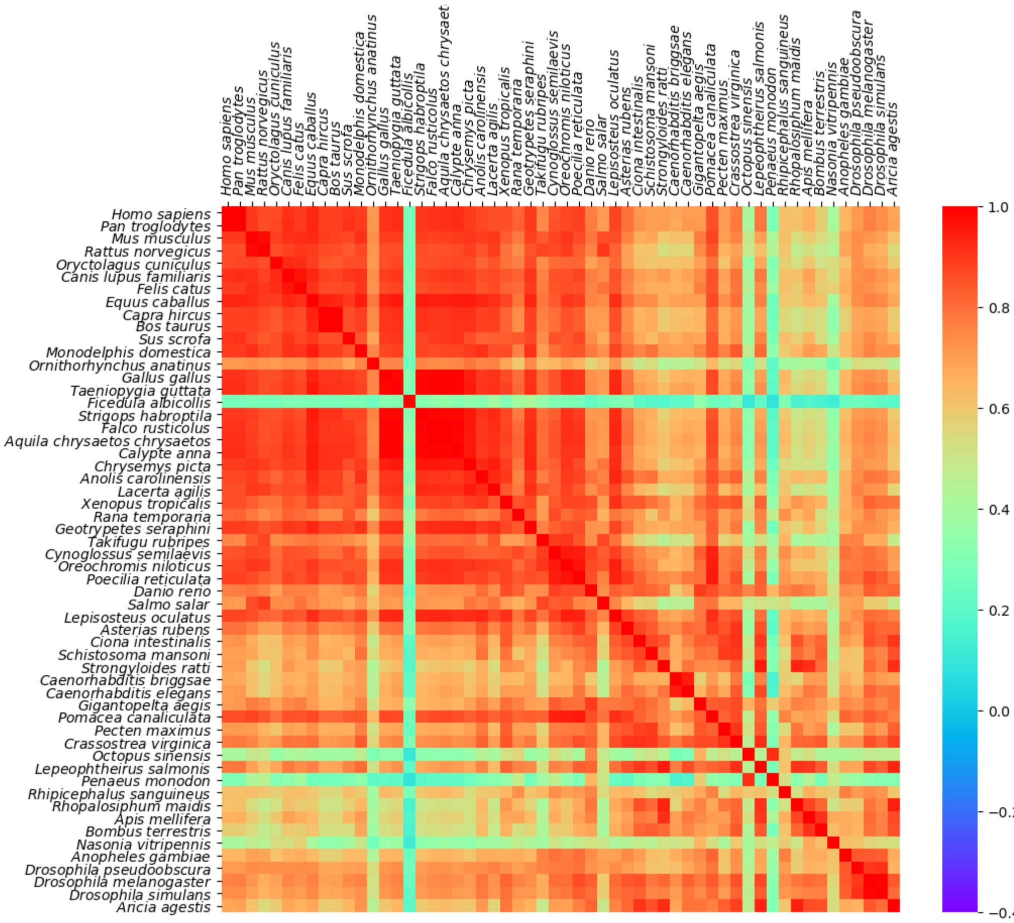

Figure S1. Heatmap of Correlations between genomes (k = 7) of *Animalia*. In each row and column the pairwise correlation values (Pearson correlation) of genomes for the listed organism are given.

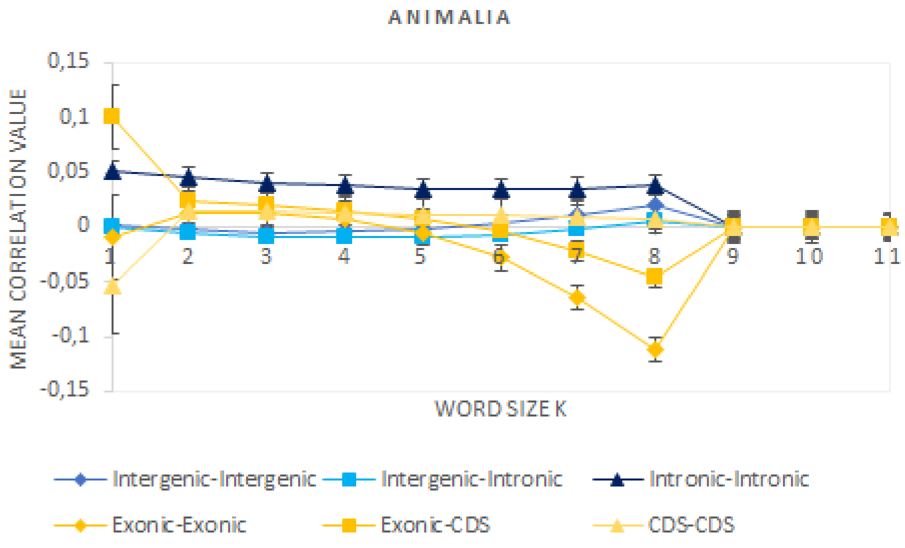

Figure S2. Mean correlation values of the difference between genomic data for the set of 56 Animalia (with 18 new genomes) and for the set of 38 Animalia against word length k (1 ≤ k ≤ 11). The error bars show the combined errors of both genomic data sets. The connecting lines between points were added for better readability.

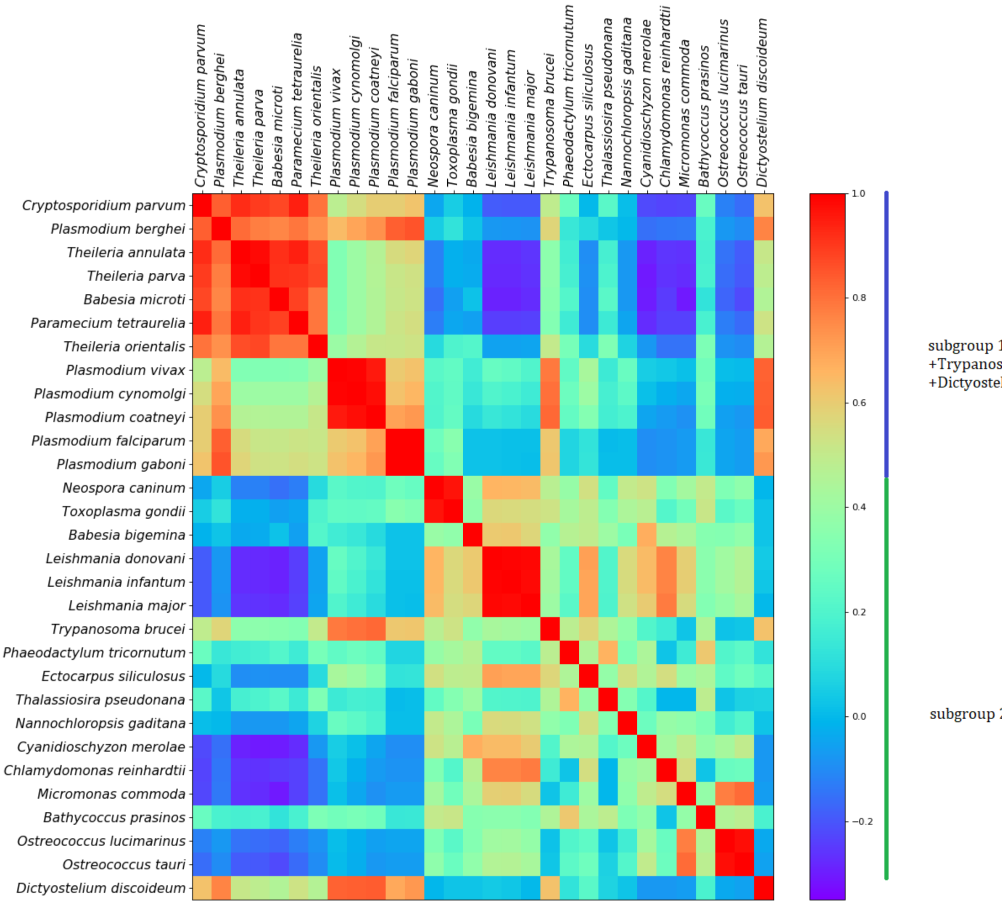

Figure S3. Heatmap of Correlations between genomes (k = 7) of Protista as a basis for the subsets. In each row and column the pairwise correlation values (Pearson correlation) of genomes for the listed organism are given.

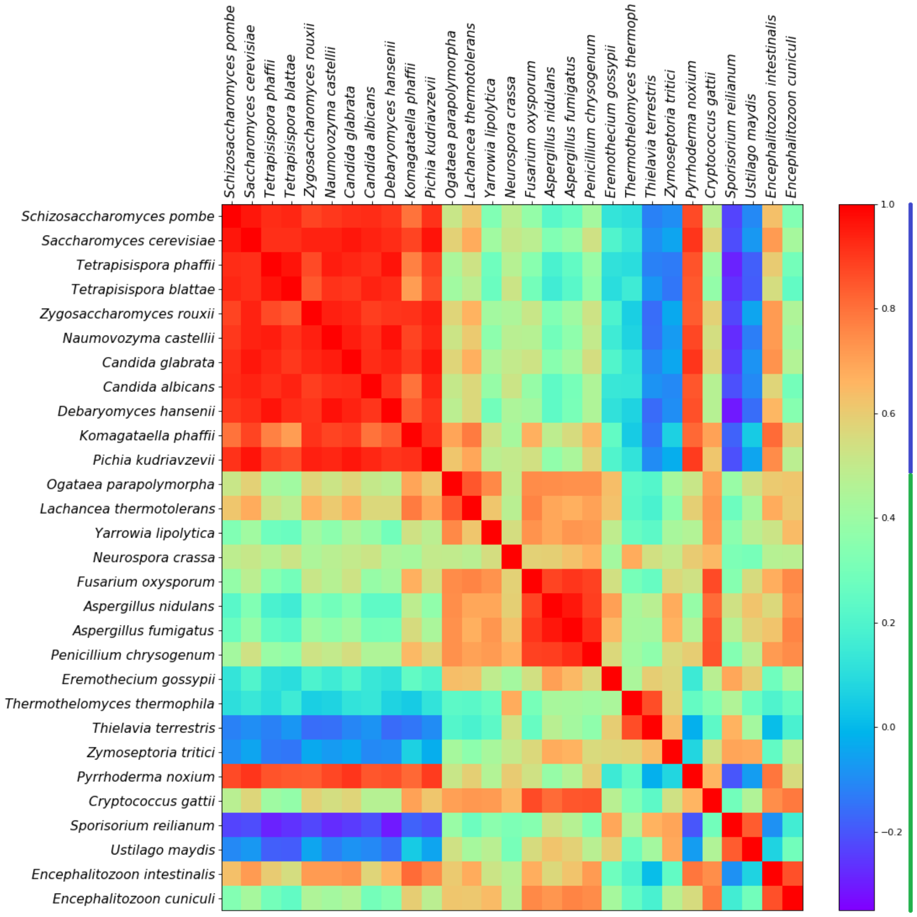

Figure S4. Heatmap of Correlations between genomes (k = 7) of Fungi as a basis for the subsets. In each row and column the pairwise correlation values (Pearson correlation) of genomes for the listed organism are given.

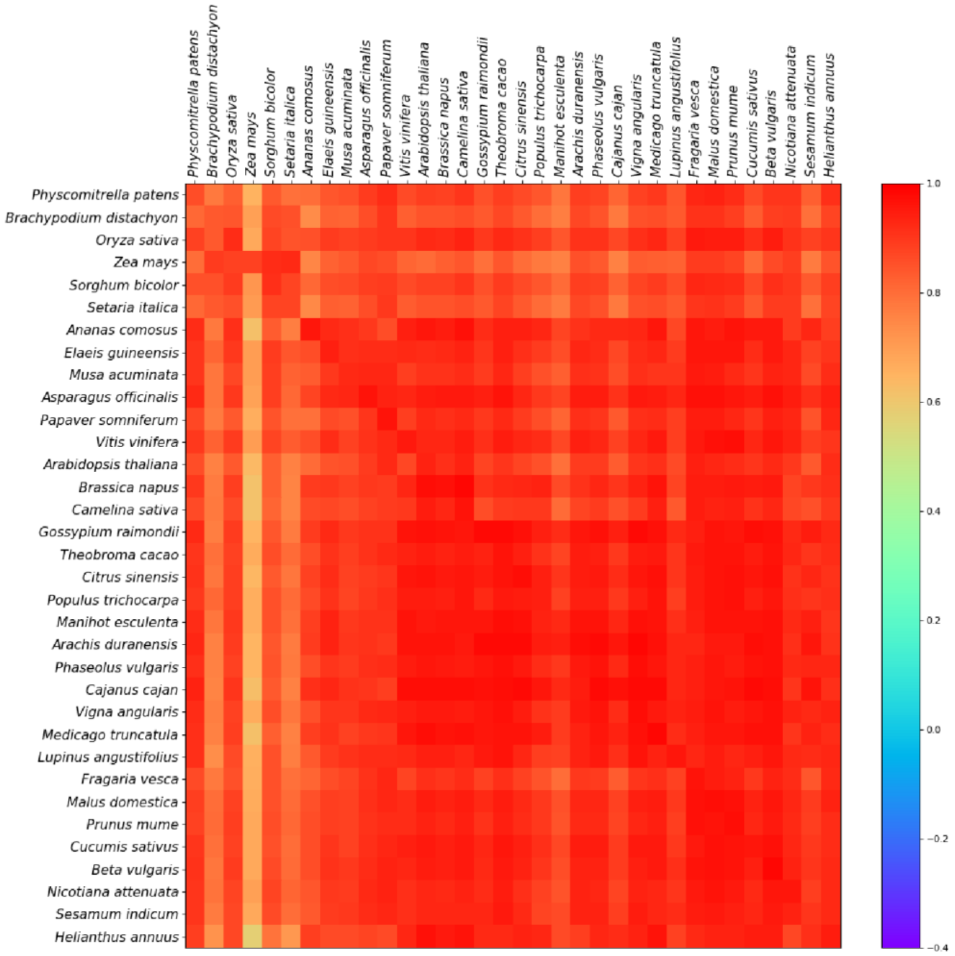

Figure S5. Heatmap of correlations between individual intron and intergenic regions (k = 7) of Embryophyta. Each row represents the pairwise correlation values (Pearson correlation) of introns for the listed organism, while every column is associated with the intergenic region of an organism. Color scale is limited in the heatmap to values above -0.4 for better readability.

Table S6. k-words and contributions of ten most contributing words for (E) Embryophyta, (P) Protista, (F) Fungi and (A) Animalia . Under "Regions" E=Exonic, I=Intronic, Int=Intergenic

Regions	*k*-word (E)	Contrib.	*k*-word (P)	Contrib.	*k*-word (F)	Contrib.	*k*-word (A)	Contrib.
**E – E**	AAAAAAA	0.0073	AAAAAAA	0.0124	GAAGAAG	0.0062	TTTTTTT	0.0204
	TTTTTTT	0.0073	TTTTTTT	0.0124	CTTCTTC	0.0061	AAAAAAA	0.0196
	TCTTCTT	0.0053	CTTCTTC	0.0056	AAGAAGA	0.0056	GCTGCTG	0.0045
	CTTCTTC	0.0053	GAAGAAG	0.0056	TCTTCTT	0.0055	CAGCAGC	0.0044
	AAGAAGA	0.0053	GGCGGCG	0.0051	AGAAGAA	0.0044	CTGCTGC	0.0032
	GAAGAAG	0.0053	CGCCGCC	0.0049	TTCTTCT	0.0044	GCAGCAG	0.0032
	TTCTTCT	0.0050	CAGCAGC	0.0040	TGAAGAA	0.0026	TTTTCTT	0.0029
	AGAAGAA	0.0049	GCGGCGG	0.0040	TTCTTCA	0.0025	TTTCTTT	0.0028
	TCTCTCT	0.0041	CGGCGGC	0.0039	GCTGCTG	0.0023	TTTATTT	0.0028
	AGAGAGA	0.0041	CCGCCGC	0.0039	CAGCAGC	0.0023	TGCTGCT	0.0027
**CDS – CDS**	CTTCTTC	0.0061	AAAAAAA	0.0103	GAAGAAG	0.0064	GCTGCTG	0.0077
	GAAGAAG	0.0061	TTTTTTT	0.0102	CTTCTTC	0.0064	CAGCAGC	0.0077
	TCTTCTT	0.0052	CTTCTTC	0.0060	AAGAAGA	0.0057	CTGCTGC	0.0054
	AAGAAGA	0.0052	GAAGAAG	0.0060	TCTTCTT	0.0056	GCAGCAG	0.0053
	AGAAGAA	0.0046	GGCGGCG	0.0055	AGAAGAA	0.0044	TGCTGCT	0.0042
	TTCTTCT	0.0046	CGCCGCC	0.0052	TTCTTCT	0.0044	AGCAGCA	0.0041
	TTCTTCA	0.0033	GCGGCGG	0.0043	TGAAGAA	0.0026	CTCCTCC	0.0035
	TGAAGAA	0.0033	CGGCGGC	0.0042	TTCTTCA	0.0025	GGAGGAG	0.0035
	CTCCTCC	0.0023	CCGCCGC	0.0041	CAGCAGC	0.0024	CTTCTTC	0.0031
	GGAGGAG	0.0023	CAGCAGC	0.0041	GCTGCTG	0.0024	GAAGAAG	0.0030
**I -I**	AAAAAAA	0.0414	TTTTTTT	0.0952	AAAAAAA	0.0683	TTTTTTT	0.0553
	TTTTTTT	0.0411	AAAAAAA	0.0929	TTTTTTT	0.0664	AAAAAAA	0.0552
	ATATATA	0.0098	TATATAT	0.0641	TACTAAC	0.0261	TGTGTGT	0.0070
	TATATAT	0.0098	ATATATA	0.0639	GTTAGTA	0.0241	ACACACA	0.0070
	AAAAAAT	0.0063	TGTGTGT	0.0111	ACTAACA	0.0137	TTTATTT	0.0065
	ATTTTTT	0.0063	GTGTGTG	0.0090	TGTTAGT	0.0129	AAATAAA	0.0064
	TATTTTT	0.0054	ACACACA	0.0061	GTATGTT	0.0060	ATATATA	0.0063
	AAAAATA	0.0053	GCGCGCG	0.0058	TTACTAA	0.0052	TATATAT	0.0063
	AAAATAA	0.0050	AAATAAA	0.0058	AACATAC	0.0048	ATTTTTT	0.0056
	TTATTTT	0.0050	CGCGCGC	0.0057	TTAGTAA	0.0045	GTGTGTG	0.0056
**Int – Int**	AAAAAAA	0.0392	TTTTTTT	0.1119	AAAAAAA	0.0669	TTTTTTT	0.0467
	TTTTTTT	0.0389	AAAAAAA	0.1112	TTTTTTT	0.0660	AAAAAAA	0.0466
	ATATATA	0.0167	TATATAT	0.0382	TATATAT	0.0152	ATATATA	0.0073
	TATATAT	0.0167	ATATATA	0.0381	ATATATA	0.0150	TATATAT	0.0072
	ATTTTTT	0.0087	TGTGTGT	0.0075	AAAAAAT	0.0055	AAATAAA	0.0069
	AAAAAAT	0.0087	ACACACA	0.0071	AAATAAA	0.0053	TTTATTT	0.0069
	TATTTTT	0.0076	GTGTGTG	0.0070	TTTATTT	0.0051	TGTGTGT	0.0059
	AAAAATA	0.0076	CACACAC	0.0067	ATTTTTT	0.0051	ACACACA	0.0057
	AAAATAA	0.0072	ATTTTTT	0.0065	TTTCTTT	0.0043	ATTTTTT	0.0055
	TTATTTT	0.0072	AAAAAAT	0.0064	AAAGAAA	0.0042	AAAAAAT	0.0055
**I – Int**	AAAAAAA	0.0416	AAAAAAA	0.1047	AAAAAAA	0.0848	TTTTTTT	0.0510
	TTTTTTT	0.0413	TTTTTTT	0.1042	TTTTTTT	0.0830	AAAAAAA	0.0510
	ATATATA	0.0135	TATATAT	0.0640	AAAAAAT	0.0055	ATATATA	0.0068
	TATATAT	0.0135	ATATATA	0.0635	ATTTTTT	0.0053	TATATAT	0.0068

	AAAAAAT	0.0076	AAATAAA	0.0075	TATATAT	0.0053	AAATAAA	0.0066
	ATTTTTT	0.0076	TTTATTT	0.0072	TTTCTTT	0.0051	TTTATTT	0.0066
	AAATAAA	0.0064	AAAAATA	0.0061	ATATATA	0.0050	TGTGTGT	0.0064
	TTTATTT	0.0063	TTATTTT	0.0061	TTTTCTT	0.0045	ACACACA	0.0063
	AAAATAA	0.0062	TATTTTT	0.0060	AAAGAAA	0.0044	ATTTTTT	0.0056
	TTATTTT	0.0062	AATAATA	0.0059	GAAAAAA	0.0043	AAAAAAT	0.0056
**E – I**	AAAAAAA	0.0254	TTTTTTT	0.0583	AAAAAAA	0.0169	TTTTTTT	0.0414
	TTTTTTT	0.0244	AAAAAAA	0.0571	TTTTTTT	0.0162	AAAAAAA	0.0406
	TTTTCTT	0.0055	ATATATA	0.0220	TCTTCTT	0.0071	TTTATTT	0.0052
	AAGAAAA	0.0055	TATATAT	0.0218	AAGAAGA	0.0070	AAATAAA	0.0047
	AAAGAAA	0.0051	AAATAAA	0.0081	AGAAGAA	0.0064	ATTTTTT	0.0044
	TTTCTTT	0.0050	AAAAAAT	0.0078	GAAGAAG	0.0064	TTTAAAA	0.0042
	AAAAGAA	0.0041	TTTATTT	0.0076	CTTCTTC	0.0060	TTTTAAA	0.0042
	TTCTTTT	0.0041	ATTTTTT	0.0075	TTCTTTT	0.0055	AAAAAAT	0.0041
	TATATAT	0.0038	AAAAATA	0.0070	TTTCTTT	0.0055	ACACACA	0.0040
	ATATATA	0.0037	TATTTTT	0.0069	TTTTCTT	0.0053	TGTGTGT	0.0039
**E – CDS**	CTTCTTC	0.0060	AAAAAAA	0.0113	GAAGAAG	0.0060	GCTGCTG	0.0065
	GAAGAAG	0.0059	TTTTTTT	0.0112	CTTCTTC	0.0060	CAGCAGC	0.0065
	TCTTCTT	0.0055	CTTCTTC	0.0055	AAGAAGA	0.0054	CTGCTGC	0.0047
	AAGAAGA	0.0054	GAAGAAG	0.0055	TCTTCTT	0.0053	GCAGCAG	0.0046
	TTCTTCT	0.0050	GGCGGCG	0.0053	AGAAGAA	0.0042	TGCTGCT	0.0037
	AGAAGAA	0.0050	CGCCGCC	0.0051	TTCTTCT	0.0042	AGCAGCA	0.0037
	TTCTTCA	0.0030	GCGGCGG	0.0042	TGAAGAA	0.0025	GGAGGAG	0.0030
	TGAAGAA	0.0030	CGGCGGC	0.0041	TTCTTCA	0.0024	CTCCTCC	0.0030
	TTTCTTC	0.0024	CCGCCGC	0.0040	CAGCAGC	0.0023	TTCTTCT	0.0025
	GAAGAAA	0.0024	GCCGCCG	0.0040	GCTGCTG	0.0023	AGAAGAA	0.0025

Table S7. Correlation Contributions of different G+C content (k = 7). Shown are the correlation contributions in % for correlations between different genome regions for k-mer words with different portions of G+C for k = 7, (E/P/F) (*Embryophyta/Protista/Fungi*).

Correlated Regions	0% G+C (E/P/F)	0% - 25% G+C (E/P/F)	25% - 50% G+C (E/P/F)	50% - 75% G+C (E/P/F)	75% - 100% G+C (E/P/F)	100% G+C (E/P/F)
**Intergenic – Intergenic**	43.86/50.48/37.36/	26.13/11.02/24.59	16.08/19.43/19.62	11.58/14.48/14.67	2.08/2.31/3.26	0.26/2.28/0.50
**Intergenic – Intronic**	38.15/68.34/32.91	28.96/10.82/24.23	17.46/9.97/21.21	12.57/8.49/17.12	2.52/1.09/3.90	0.35/1.29/0.63
**Intronic – Intronic**	31.32/53.22/21.32	31.10/10.92/20.02	20.58/17.42/39.00	13.71/13.82/16.15	2.87/2.35/3.01	0.42/2.27/0.49
**Exonic – Intronic**	14.32/49.57/7.15	24.18/16.28/14.88	38.09/16.29/49.31	19.53/13.91/26.01	3.59/2.22/2.27	0.29/1.73/0.37
**Exonic – Exonic**	3.55/12.60/1.03	11.71/10.14/8.48	53.51/35.59/56.41	27.36/30.54/31.32	3.01/6.86/2.21	0.87/4.27/0.56
**Exonic – CDS**	0.48/12.50/1.11	8.01/10.31/8.69	57.55/34.44/55.21	29.90/30.62/31.65	2.95/7.50/2.63	1.11/4.63/0.72
**CDS – CDS**	0.24/11.73/0.73	5.91/9.67/7.87	58.86/35.66/56.59	31.03/31.26/32.00	2.84/7.16/2.21	1.12/4.52/0.59

Table S8. Correlation Contributions of different G+C content (k = 11). Shown are the correlation contributions in % for correlations between different genome regions for k-mer words with different portions of G+C for k = 11, (E/P/F) (*Embryophyta/Protista/Fungi*).

Correlated Regions	0% G+C (E/P/F)	0% - 25% G+C (E/P/F)	25% - 50% G+C (E/P/F)	50% - 75% G+C (E/P/F)	75% - 100% G+C (E/P/F)	100% G+C (E/P/F)
**Intergenic – Intergenic**	59.62/59.62/50.92	25.14/12.32/28.31	10.20/12.51/13.06	4.68/11.79/6.64	0.25/2.05/0.58	0.11/1.71/0.49
**Intergenic – Intronic**	52.53/76.85/50.92	28.07/8.85/25.47	12.96/5.81/13.67	5.91/5.78/8.05	0.34/1.55/0.74	0.19/1.16/1.14
**Intronic – Intronic**	43.40/68.08/45.39	31.33/11.84/23.87	17.28/8.60/19.90	7.22/7.70/8.20	0.44/2.27/0.72	0.31/1.50/1.93
**Exonic – Intronic**	22.29/63.68/14.36	24.63/16.83/16.51	36.98/7.51/42.89	15.25/7.66/24.67	0.62/2.28/0.92	0.23/2.04/0.65
**Exonic – Exonic**	5.66/12.85/0.89	10.49/13.78/9.02	55.17/26.56/53.63	25.5732.59/34.03	1.61/9.84/1.99	1.50/4.38/0.44
**Exonic – CDS**	0.27/12.08/0.61	5.93/13.20/8.85	61.57/26.59/52.86	27.41/33.00/34.84	2.42/10.30/2.33	2.40/4.82/0.51
**CDS – CDS**	0.04/11.40/0.45	3.92/13.38/8.01	62.44/27.07/53.58	28.88/33.50/35.31	2.48/10.13/2.16	2.24/4.53/0.49

Table S9. Correlation Contributions of tandem repeat words. Shown are the correlation contributions for correlations between introns and intergenic regions of the subsets in Protista and in Fungi of for tandem repeat k-mer words with repeat unit length ≤ 2bp for no mismatch allowed for k = 7 (top) and k = 11 (bottom). In rows with no mm dark green if in top 4, light green if in top 16, bright green if in top 128, orange if in top 1600, red if in rest

k-mer word	Protista #S1	Protista #S2	Fungi #S1	Fungi #S2
AAAAAAA	13.69%	0.76%	7.17%	7.44%
CCCCCCC	< 0.01%	0.83%	< 0.01%	0.13%
GGGGGGG	< 0.01%	0.76%	< 0.01%	0.14%
TTTTTTT	13.69%	0.81%	7.24%	7.04%
ACACACA	0.03%	1.93%	0.01%	0.06%
AGAGAGA	< 0.01%	1.04%	< 0.01%	0.37%
ATATATA	4.54%	1.46%	0.43%	0.43%
CACACAC	0.02%	1.97%	< 0.01%	0.06%
CGCGCGC	< 0.01%	2.11%	< 0.01%	<0.01%
CTCTCTC	< 0.01%	0.99%	< 0.01%	0.30%
GAGAGAG	< 0.01%	1.00%	< 0.01%	0.31%
GCGCGCG	< 0.01%	2.14%	< 0.01%	<0.01%
GTGTGTG	0.02%	2.60%	< 0.01%	0.07%
TATATAT	4.57%	1.46%	0.46%	0.44%
TCTCTCT	< 0.01%	1.06%	0.01%	0.37%
TGTGTGT	0.04%	2.60%	< 0.01%	0.08%
AAAAAAAAAAA	21.18%	0.10%	10.87%	28.36%
CCCCCCCCCCC	< 0.01%	1.50%	0.03%	1.41%
GGGGGGGGGGG	< 0.01%	1.51%	0.05%	1.25%
TTTTTTTTTTT	21.07%	0.11%	9.12%	26.34%
ACACACACACA	< 0.01%	7.98%	0.07%	0.18%
AGAGAGAGAGA	< 0.01%	1.97%	0.02%	0.98%
ATATATATATA	7.27%	6.05%	1.16%	0.28%
CACACACACAC	0.01%	8.04%	0.06%	0.19%
CGCGCGCGCGC	< 0.01%	1.25%	< 0.01%	<0.01%
CTCTCTCTCTC	< 0.01%	1.92%	0.03%	0.78%
GAGAGAGAGAG	< 0.01%	1.79%	0.01%	0.97%
GCGCGCGCGCG	< 0.01%	1.25%	< 0.01%	<0.01%
GTGTGTGTGTG	< 0.01%	9.47%	0.01%	0.24%
TATATATATAT	7.35%	6.08%	1.36%	0.29%
TCTCTCTCTCT	< 0.01%	2.23%	0.03%	0.78%
TGTGTGTGTGT	< 0.01%	9.42%	0.02%	0.23%

Table S10. Preferences of nucleotides within mismatches of tandem repeat words. Contents of specific nucleotides (A, C, G, T) within mismatches of tandem repeat k-mer words in Embryophyta (for k = 7 and k = 11). Contents lower than 20% are marked red, and contents higher than 30% are marked green, remaining contents are marked orange.

	Intronic Region				Intergenic Region			
***k*-mer word**	**A**	**C**	**G**	**T**	**A**	**C**	**G**	**T**
AAAAAAA	-	28.25%	32.25%	39.50%	-	23.34%	28.20%	48.47%
CCCCCCC	39.79%	-	12.52%	47.69%	38.78%	-	14.76%	46.46%
GGGGGGG	47.80%	12.25%	-	39.94%	46.49%	14.75%	-	38.75%
TTTTTTT	39.53%	32.20%	28.27%	-	48.45%	28.22%	23.33%	-
ACACACA	52.88%	12.29%	12.09%	22.74%	48.34%	15.31%	11.27%	25.09%
AGAGAGA	53.26%	11.13%	15.85%	19.76%	49.96%	10.33%	18.27%	21.44%
ATATATA	37.19%	15.64%	16.07%	31.10%	39.30%	13.49%	13.65%	33.56%
CACACAC	47.60%	14.28%	12.27%	25.86%	43.40%	18.50%	11.74%	26.36%
CGCGCGC	30.72%	21.25%	19.93%	28.11%	27.13%	24.45%	22.06%	26.36%
CTCTCTC	21.28%	19.21%	11.76%	47.75%	22.56%	21.20%	11.09%	45.15%
GAGAGAG	47.69%	11.73%	19.28%	21.31%	45.21%	11.08%	21.18%	22.53%
GCGCGCG	28.05%	19.94%	21.38%	30.63%	26.32%	22.16%	24.14%	27.37%
GTGTGTG	25.93%	12.23%	14.37%	47.46%	26.34%	11.75%	18.52%	43.39%
TATATAT	31.12%	16.07%	15.67%	37.14%	33.56%	13.66%	13.48%	39.30%
TCTCTCT	19.79%	15.79%	11.17%	53.25%	21.49%	18.31%	10.33%	49.87%
TGTGTGT	22.81%	12.08%	12.35%	52.76%	25.12%	11.27%	15.28%	48.34%
Mean	36.36%	16.95%	17.02%	36.33%	36.16%	17.19%	17.15%	36.16%
AAAAAAAAAAA	-	28.15%	37.88%	33.97%	-	23.22%	33.48%	43.30%
CCCCCCCCCCC	41.91%	-	12.25%	45.83%	37.92%	-	13.14%	48.94%
GGGGGGGGGGG	46.58%	11.70%	-	41.72%	49.07%	12.96%	-	37.97%
TTTTTTTTTTT	33.99%	37.78%	28.23%	-	43.28%	33.51%	23.21%	-
ACACACACACA	43.28%	12.14%	15.63%	28.95%	40.12%	14.42%	13.65%	31.81%
AGAGAGAGAGA	45.41%	10.86%	25.69%	18.04%	48.47%	8.48%	25.02%	18.03%
ATATATATATA	27.95%	20.45%	20.25%	31.35%	30.36%	17.35%	17.38%	34.91%
CACACACACAC	42.55%	11.07%	15.38%	30.99%	39.78%	14.59%	13.53%	32.09%
CGCGCGCGCGC	37.24%	12.15%	16.27%	34.34%	29.30%	23.46%	19.84%	27.40%
CTCTCTCTCTC	18.06%	24.86%	11.01%	46.07%	18.79%	24.71%	8.56%	47.94%
GAGAGAGAGAG	45.58%	11.08%	25.09%	18.25%	48.07%	8.55%	24.78%	18.60%
GCGCGCGCGCG	34.51%	13.57%	16.80%	35.12%	27.57%	20.43%	21.45%	30.55%
GTGTGTGTGTG	30.89%	15.58%	11.35%	42.19%	32.11%	13.59%	14.43%	39.87%
TATATATATAT	31.33%	20.50%	20.19%	27.98%	35.05%	17.40%	17.27%	30.28%
TCTCTCTCTCT	17.94%	25.58%	10.78%	45.70%	18.22%	24.94%	8.49%	48.35%
TGTGTGTGTGT	29.06%	15.75%	12.27%	42.93%	31.75%	13.74%	14.32%	40.19%
Mean	35.09%	18.08%	18.61%	34.90%	35.32%	18.09%	17.90%	35.35%

Table S11. Preferences of nucleotides within mismatches of tandem repeat words. Contents of specific nucleotides (A, C, G, T) within mismatches of tandem repeat k-mer words in Protista (for k = 7 and k = 11). Contents lower than 20% are marked red, and contents higher than 30% are marked green, remaining contents are marked orange.

k-mer word	Intronic Region A	C	G	T	Intergenic Region A	C	G	T
AAAAAAA	-	31.12%	35.81%	33.07%	-	32.04%	38.56%	29.40%
CCCCCCC	30.42%	-	24.94%	44.64%	29.76%	-	25.66%	44.57%
GGGGGGG	44.90%	26.09%	-	29.01%	44.58%	25.78%	-	29.65%
TTTTTTT	32.99%	36.13%	30.88%	-	29.02%	38.51%	32.47%	-
ACACACA	36.86%	16.18%	22.63%	24.33%	33.28%	18.31%	26.44%	21.98%
AGAGAGA	41.13%	17.44%	26.54%	14.89%	39.91%	19.60%	26.01%	14.48%
ATATATA	35.03%	19.42%	20.48%	25.07%	29.67%	22.36%	23.40%	24.56%
CACACAC	28.68%	19.33%	23.77%	28.21%	29.26%	20.14%	26.82%	23.78%
CGCGCGC	29.06%	22.09%	19.26%	29.59%	29.56%	20.61%	20.48%	29.35%
CTCTCTC	15.31%	29.67%	17.78%	37.24%	14.80%	31.21%	20.03%	33.96%
GAGAGAG	36.07%	17.82%	30.62%	15.49%	34.43%	20.07%	30.64%	14.85%
GCGCGCG	29.77%	18.14%	21.94%	30.15%	29.80%	20.27%	20.85%	29.09%
GTGTGTG	27.32%	23.18%	18.01%	31.49%	23.99%	26.80%	19.72%	29.49%
TATATAT	25.79%	20.22%	19.42%	34.57%	24.44%	23.17%	22.32%	30.07%
TCTCTCT	14.23%	24.29%	17.75%	43.73%	14.37%	26.53%	19.60%	39.49%
TGTGTGT	24.77%	23.19%	15.68%	36.36%	22.03%	26.60%	17.98%	33.39%
Mean	30.16%	22.95%	23.03%	30.52%	28.59%	24.80%	24.73%	28.54%
AAAAAAAAAAA	-	30.83%	36.60%	32.56%	-	34.67%	38.21%	27.12%
CCCCCCCCCCC	24.53%	-	26.18%	49.29%	25.61%	-	26.38%	48.01%
GGGGGGGGGGG	50.28%	27.32%	-	22.41%	47.20%	26.30%	-	26.50%
TTTTTTTTTTT	30.86%	37.06%	32.07%	-	25.74%	38.68%	35.58%	-
ACACACACACA	33.68%	12.97%	21.38%	31.96%	28.90%	17.02%	29.14%	24.93%
AGAGAGAGAGA	37.98%	15.17%	33.88%	12.97%	38.47%	19.75%	28.71%	13.07%
ATATATATATA	26.18%	24.30%	25.35%	24.17%	22.66%	24.17%	26.53%	26.65%
CACACACACAC	33.34%	14.31%	18.86%	33.49%	30.62%	14.45%	27.92%	27.01%
CGCGCGCGCGC	24.54%	25.20%	25.25%	25.00%	31.14%	18.31%	22.88%	27.68%
CTCTCTCTCTC	12.55%	34.56%	17.22%	35.67%	12.05%	32.07%	20.11%	35.77%
GAGAGAGAGAG	36.65%	16.71%	35.42%	11.23%	36.85%	19.94%	30.80%	12.41%
GCGCGCGCGCG	27.20%	22.85%	24.40%	25.55%	28.89%	20.98%	21.30%	28.82%
GTGTGTGTGTG	32.26%	22.13%	11.82%	33.79%	26.57%	28.46%	14.42%	30.55%
TATATATATAT	28.05%	25.64%	24.75%	21.56%	24.84%	27.78%	23.32%	24.05%
TCTCTCTCTCT	12.06%	31.75%	17.02%	39.17%	12.15%	29.68%	19.23%	38.94%
TGTGTGTGTGT	29.81%	22.86%	12.77%	34.57%	23.86%	28.84%	16.12%	31.19%
Mean	29.33%	24.24%	24.20%	28.89%	27.70%	25.41%	25.38%	28.18%

Table S12. Preferences of nucleotides within mismatches of tandem repeat words. Contents of specific nucleotides (A, C, G, T) within mismatches of tandem repeat k-mer words in Fungi (for k = 7 and k = 11). Contents lower than 20% are marked red, and contents higher than 30% are marked green, remaining contents are marked orange.

k-mer word	Intronic Region A	C	G	T	Intergenic Region A	C	G	T
AAAAAAA	-	29.79%	40.33%	29.88%	-	27.21%	38.86%	33.94%
CCCCCCC	32.65%	-	21.27%	46.08%	33.71%	-	22.37%	43.92%
GGGGGGG	49.26%	20.37%	-	30.37%	44.49%	22.65%	-	32.85%
TTTTTTT	29.41%	41.20%	29.39%	-	33.82%	38.70%	27.48%	-
ACACACA	40.76%	15.74%	19.07%	24.42%	38.75%	18.08%	19.32%	23.85%
AGAGAGA	43.01%	17.46%	20.89%	18.63%	42.57%	17.83%	19.50%	20.11%
ATATATA	27.65%	25.84%	21.76%	24.75%	31.76%	19.49%	19.65%	29.10%
CACACAC	31.19%	14.30%	18.53%	35.98%	33.17%	20.60%	20.26%	25.97%
CGCGCGC	28.83%	22.00%	18.78%	30.40%	26.98%	23.01%	22.64%	27.38%
CTCTCTC	20.65%	21.13%	18.71%	39.52%	20.80%	20.53%	19.00%	39.68%
GAGAGAG	40.22%	18.14%	21.63%	20.00%	39.81%	18.85%	20.50%	20.84%
GCGCGCG	28.41%	19.27%	24.03%	28.30%	27.39%	20.60%	25.30%	26.71%
GTGTGTG	35.39%	18.13%	15.80%	30.68%	25.57%	20.58%	20.62%	33.24%
TATATAT	23.25%	22.59%	25.45%	28.72%	28.92%	19.65%	19.32%	32.11%
TCTCTCT	18.74%	19.65%	17.02%	44.58%	20.09%	19.36%	18.13%	42.42%
TGTGTGT	24.72%	18.81%	15.60%	40.87%	23.66%	19.73%	17.88%	38.73%
Mean	31.61%	21.63%	21.88%	31.55%	31.43%	21.79%	22.06%	31.39%
AAAAAAAAAAA	-	25.81%	45.27%	28.92%	-	24.45%	45.46%	30.09%
CCCCCCCCCCC	28.28%	-	13.17%	58.56%	29.17%	-	19.84%	50.99%
GGGGGGGGGGG	56.81%	17.70%	-	25.49%	53.15%	18.67%	-	28.18%
TTTTTTTTTTT	23.76%	50.62%	25.61%	-	30.58%	44.19%	25.23%	-
ACACACACACA	34.99%	18.58%	21.78%	24.65%	27.23%	22.11%	23.19%	27.47%
AGAGAGAGAGA	47.65%	13.10%	29.65%	9.61%	46.16%	14.78%	24.27%	14.79%
ATATATATATA	23.52%	25.65%	22.04%	28.80%	28.46%	20.48%	21.29%	29.77%
CACACACACAC	30.74%	15.63%	26.78%	26.85%	27.42%	22.87%	23.02%	26.69%
CGCGCGCGCGC	25.18%	21.90%	23.32%	29.60%	26.35%	23.81%	20.75%	29.10%
CTCTCTCTCTC	11.72%	30.59%	16.23%	41.45%	14.68%	25.16%	15.43%	44.74%
GAGAGAGAGAG	45.67%	13.48%	30.57%	10.29%	44.54%	15.14%	24.83%	15.49%
GCGCGCGCGCG	21.17%	19.86%	33.31%	25.66%	25.54%	24.34%	21.68%	28.45%
GTGTGTGTGTG	30.68%	17.28%	21.22%	30.82%	28.54%	22.28%	20.81%	28.37%
TATATATATAT	29.24%	21.94%	22.69%	26.13%	31.44%	20.78%	20.31%	27.47%
TCTCTCTCTCT	11.98%	30.68%	14.56%	42.78%	14.83%	25.73%	15.52%	43.92%
TGTGTGTGTGT	25.03%	19.16%	22.64%	33.17%	27.64%	22.59%	20.11%	29.66%
Mean	29.76%	22.80%	24.59%	29.52%	30.38%	23.16%	22.78%	30.35%
